# High variability of plasmid uptake rates in *Escherichia coli* isolated from sewage and river sediments

**DOI:** 10.1371/journal.pone.0232130

**Published:** 2020-04-30

**Authors:** Stefanie Heß, Teppo Hiltunen, Thomas U. Berendonk, David Kneis

**Affiliations:** 1 Department of Microbiology, University of Helsinki, Helsinki, Finland; 2 Department of Biology, University of Turku, Turku, Finland; 3 Institute of Hydrobiology, TU Dresden, Dresden, Germany; 4 Helmholtz-Centre for Environmental Research, Magdeburg, Germany; Centre for Ecology and Hydrology, UNITED KINGDOM

## Abstract

The horizontal transfer of plasmids is a key mechanism behind the spread of antibiotic resistance in bacteria. So far, transfer rate constants were measured for a variety of plasmids, donors and recipients. The employed strains typically had a long history in laboratories. Existing data are, therefore, not necessarily representative for real-world environments. Moreover, information on the inter-strain variability of plasmid transfer rates is scarce. Using a high-throughput approach, we studied the uptake of RP4 by various *Escherichia coli* recipients using *Serratia marcescens* as the donor. The recipient strains were isolated from human-borne sewage and river sediments. The rate constants of plasmid transfer generally followed a log-normal distribution with considerable variance. The rate constants for good and poor recipients (95 and 5% quantile) differed by more than three orders of magnitude. Specifically, the inter-strain variability of the rate constant was large in comparison to alterations induced by low-level antibiotic exposure. We did not find evidence for diverging efficiencies of plasmid uptake between *E. coli* recipients of different origin. On average, strains isolated from river bottom sediments were equally efficient in the acquisition of RP4 as isolates extracted from sewage. We conclude that *E. coli* strains persisting in the aquatic environment and those of direct human origin share a similar intrinsic potential for the conjugative uptake of certain plasmids. In view of the large inter-strain variability, we propose to work towards probabilistic modeling of the environmental spread of antibiotic resistance.

## Introduction

Horizontal gene transfer (HGT) is known to play a vital role in the dissemination of antibiotic resistance [1–3]. The conjugative transfer of plasmids represents one of the basic mechanisms of HGT being observed in both Gram-negative and Gram-positive bacteria [4, 5]. The occurrence, mechanisms, and controls of conjugative plasmid transfer have been examined in numerous studies [6–8]. In order to better understand and eventually predict the dynamics of plasmid-mediated antibiotic resistance (AR), quantitative information on the respective transfer rates is required. Although considerable efforts have been made to quantify plasmid transfer for a variety of mating pairs [6, 7, 9, 10], the existing numerical information hardly forms a sufficient basis for predictive mechanistic modeling. One problem lies in the poor comparability between studies resulting from a lack of standardization with regard to the employed measure of the transfer efficiency [8, 11]. Fortunately, this issue has largely been solved with the adoption of a non-ambiguous definition of the transfer efficiency [12] rooted in a small set of differential equations [13]. However, several challenges remain on the way towards predictive modeling of the spread of plasmid-borne AR in real-world systems.

First of all, most studies examined plasmid transfer rates for just a single mating pair or a few particular pairs. Considering the huge genotypic and phenotypic variability occurring even at species level [14, 15], the resulting information is not necessarily representative. Moreover, the majority of reported transfer rates relates to bacterial strains with a long history of life in laboratories and many of the strains underwent genetic modification. It is hardly known whether the obtained transfer rates actually apply to natural isolates of clinical or environmental origin, for example.

With this work, we made an attempt to resolve some of these deficits taking benefit from a recently developed high-throughput technique [16]. Using the latter, we studied the variability of plasmid uptake rates among natural *Eschericha coli* isolates acting as a recipient of RP4. That specific plasmid [17] conveys a triple resistance against ampicillin, kanamycin, and tetracycline.

To capture possible disparities between strains of different origin, we chose a set of recipients (n = 96) consisting of two subsets: Half of the strains were isolated from raw sewage tanks so as to ensure a close link to the human microbiome. The other 50% comprised environmental isolates extracted from river sediments [18]. Adding to the exploration of inter-strain variabilities, we also studied the response of plasmid acquisition to sub-inhibitory levels of antibiotics.

Our results shed light on the large inter-strain variability of plasmid uptake rates which must be taken into account by predictive models.

## Material and methods

### Specification of mating pairs

Liquid phase mating experiments were carried out with *Serratia marcescens* (ATCC 1380) as the donor of plasmid RP4. The latter is a broad host range plasmid that can be acquired by most Gram-negatives and even transferred across the Gram boundary [19]. RP4 belongs to the IncP1 incompatibility group and confers resistance to three antibiotics (ampicillin, kanamycin, tetracycline). This triple-resistance is very unlikely to develop through spontaneous mutation which makes RP4 a suitable candidate for studies on conjugative gene transfer.

A total of 96 *E. coli* isolates were used as plasmid recipients. Half of them were isolated from raw sewage of human origin, namely waste tanks of airplanes and airport terminal buildings [20]). The remaining 48 isolates were extracted from the bottom sediment of a mountain stream [18]. For the purpose of *E. coli* isolation, sewage samples were diluted, plated on mFC-agar, and subsequently incubated for 18±2 h at 44°C. The same procedure was applied to the pore water of sediment samples after mobilization of particle-attached bacteria. Mobilization was achieved by shaking (20 minutes, 450 rpm) in combination with sodiumdiphosphate treatment (20 mL of 0.1% Na_4_P_2_O_7_ per 100 g fresh weight). Blue colonies were picked from the mFC plates, streaked again on Brilliance agar (Oxoid, Wesel, Germany), and grown overnight at 37°C. To identify these isolates as *E. coli*, colony PCR was performed using primers amplifying a fragment of the *ycc*T gene [21]. The detailed PCR program can be found in [18]. Amplification of the *E. coli* specific *ycc*T gene fragment was finally confirmed by agarose gelelectrophoresis.

The 96 recipients were selected from a much larger collection of isolates in consideration of antibiotic resistance profiles, the possession of plasmids, and the likelihood of independence. Specifically, none of the recipients harbored a phenotypic resistance against any of 24 common antibiotics (see [18] for list of drugs). In addition, all isolates were tested negative for IncP1 plasmids by PCR [16, 22] so as to rule out potential incompatibilities or prior infections with RP4. Finally, isolates were preferably picked from samples obtained at different locations and/or dates (17 river samples, 9 samples of sewage) such that the chance of picking clones was minimized.

### Estimation of rate constants

Mating experiments took place at 37°C in 8 × 12 deep well plates. Initially, the wells contained 600 *μL* of HT broth: a cereal grass tea amended with essential salts and the minimal medium R2A [16]. The wells were inoculated with donor and recipient (1 × 10^5^ cells mL^–1^ each). Plates were continuously shaken at 150 rpm for the whole duration of the experiment (24 h). All 96 wells were sampled synchronously at hourly intervals by means of a multi-pin replicator (EnzyScreen, Netherlands). The sub-samples of 10 *μL* per well were simultaneously transferred to Tryptone Bile X-Glucoronide (TBX) agar supplemented with 150 mg L^–1^ ampicillin, 25 mg L^–1^ kanamycin, and 20 mg L^–1^ tetracycline to select for RP4-carrying cells. The agar plates were incubated over 44–48 h at 44°C to prevent the growth of *S. marcescens*. Finally, the plates were inspected for blue spots indicating the successful formation of *E. coli* transconjugants. The resulting binary information (1 = transconjugants present, 0 = absent) was used to infer the rate constant of plasmid transfer, *γ* (mL cells^–1^ hour^–1^), by means of inverse modeling. The methodological approach was described in details previously [16].

As a prerequisite to the inference of *γ*, growth rate constants for all recipients, transconjugants, and the donor strain were derived from optical densities recorded over time (Labsystems Bioscreen C plate reader, Thermo; 600 nm wave length). Ambient conditions for the growth experiments were the same as for the conjugation assay (HT broth [16], 37°C, continuous shaking at 150 rpm).

For a subset of 16 strains (eight environmental and eight human-associated), the rate constants of growth and plasmid transfer were measured with and without exposure to tetracycline and kanamycin, including combined treatments. The tested concentrations were 0.002 and 0.02 mg L^–1^ for tetracycline and 0.0025, 0.025 and 0.25 mg L^–1^ for kanamycin. The upper limits were chosen to guarantee sub-inhibitory conditions for the recipient. Under the strongest treatment (0.02 mg L^–1^ tetracycline plus 0.25 mg L^–1^ kanamycin) the average reduction in recipient growth rates was 30% with regard to the antibiotic-free control. As concentrations were increased by another log unit, many of the recipient strains were not able to grow at all. This was especially so for treatments with 0.2 mg L^–1^ tetracycline.

Importantly, for each level of antibiotic exposure, the estimates of *γ* were obtained considering the treatment-specific growth parameters of donor, transconjugant, and recipient. All growth and conjugation experiments were repeated at least 12 times.

### Whole genome sequencing

Eight strains of each origin (four with high and four with low abilities to acquire the RP4 plasmid, respectively) were chosen and their genome sequenced using a HiSeq device (2 x 150 bp). For quality control and trimming the software TrimGalore! was used (applied specifications: q = 28, length: at least 80 bp). Subsequently, the reads were assembled with SPAdes 3.12.0 [23] and the genes were annotated using Prokka v3 [24]. The combined Prokka outputs were processed through the R function daisy from package cluster to obtain a matrix of genetic dissimilarities underlying Fig 3. The online tools from https://cge.cbs.dtu.dk/services were used to scan the genomes for plasmids [25], virulence factors [26], antibiotic resistance genes [27] and multi-locus sequence types [28]. The outputs of the latter analyses were compiled in Table 1. The sequences can be accessed through the European Nucleotide Archive ENA (accession numbers in Table 1).

**Table 1:** Plasmids, virulence factors, and antibiotic resistance genes harbored by the 16 *E. coli* recipients that underwent whole-genome sequencing.

Isolate	Origin	MLST	Plasmids	Virulence factors	Antibiotic res. genes	Accession number
1	Sediment	155	IncFIB	astA, gad, lpfA	mdf(A)	ERS4386668
2	Sediment	154	–	gad, iss, lpfA	mdf(A)	ERS4386669
3	Sediment	3549	IncFIB, IncFII	astA, f17A, f17G, gad, iss, lpfA	mdf(A)	ERS4386671
4	Sediment	1642	–	gad, lpfA	mdf(A)	ERS4386670
5	Sewage	226	IncFII, IncX3, IncY	cba, cma, gad	qnrB7, mdf(A)	ERS4386952
6	Sewage	226	IncFII, IncX3, IncY	cba, cma, gad	qnrB7, mdf(A)	ERS4386953
7	Sewage	226	IncFII, IncX3, IncY	cba, cma, gad	qnrB7, mdf(A)	ERS4386954
8	Sewage	104	–	gad, pic, vat	mdf(A)	ERS4386955
9	Sewage	1128	–	gad, iss, lpfA	mdf(A)	ERS4386956
10	Sewage	536	Col440I	gad, lpfA	mdf(A)	ERS4386957
11	Sewage	2139	Col156, IncFII, IncY	celb, gad	mdf(A)	ERS4386958
12	Sewage	130	–	aaiC, air, eilA, gad, lpfA, nfaE, pic	mdf(A)	ERS4386959
13	Sediment	1829	–	air, eilA	fosA7, mdf(A)	ERS4386672
14	Sediment	1850	–	gad, iss	mdf(A)	ERS4386673
15	Sediment	212	IncI1	gad, lpfA	mdf(A)	ERS4386674
16	Sediment	3747	IncFII (pSE11), IncY	cif, eae, espA, espF, gad, iss, tir	–	ERS4386675

Note that strain 5–7 represent the same sequence type but they are not exact clones according to their complete gene inventory. MLST: Multi-locus sequence type. Accession numbers refer to the European Nucleotide Archive (ENA).

## Results

### Intrinsic growth rates of the *E. coli* isolates

**Fig 1:**
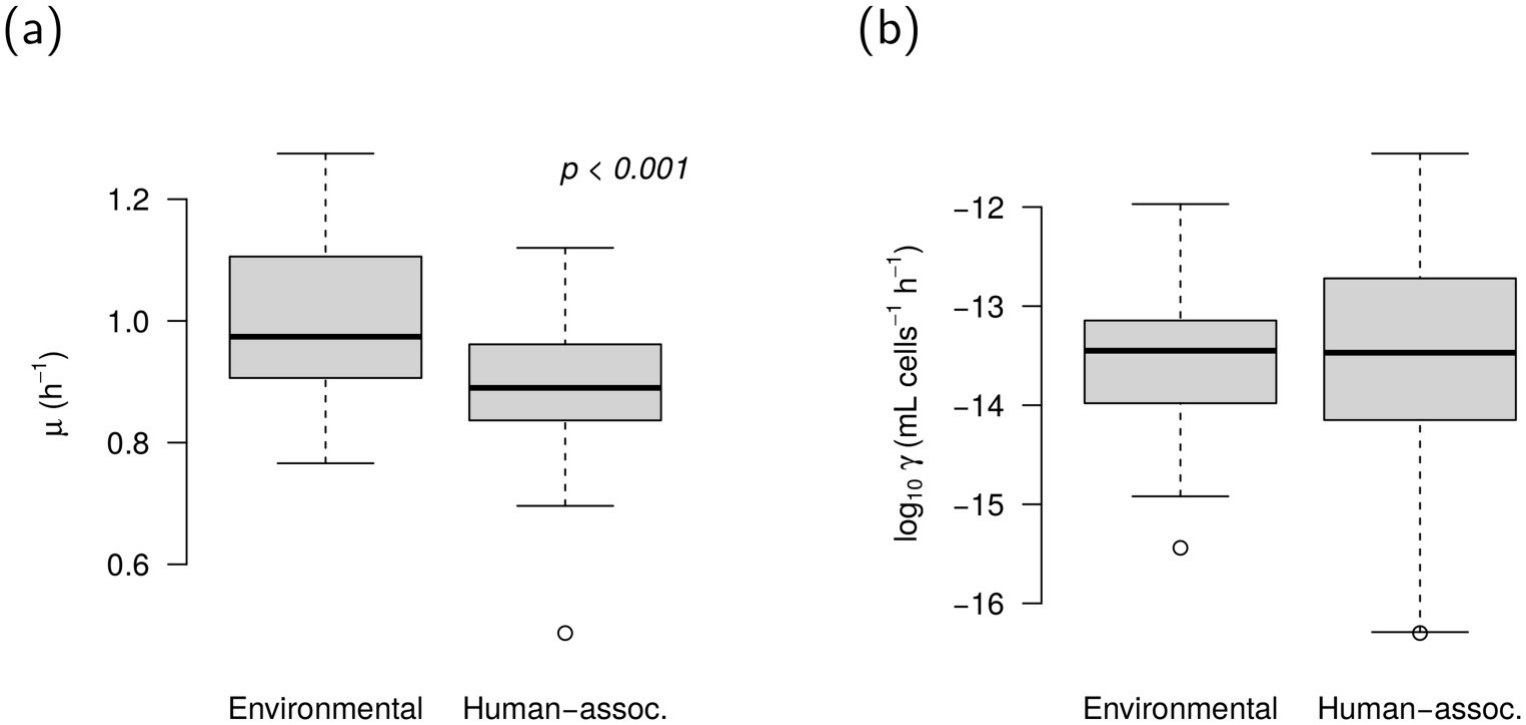
Characteristics of *E. coli* strains isolated from river sediments and human-borne raw sewage. a: Intrinsic growth rate constants. The *p*-value indicates a significant shift in location (U test). b: Bulk conjugation rate constants. All data refer to the case without antibiotic exposure. Boxes depict interquartile ranges, whiskers extend to the most extreme data points not being classified as outliers according to the conventional 1.5 × IQR rule. Outliers are displayed as dots.

The two subsets of *E. coli* isolates differed significantly with regard to their intrinsic growth rates (Fig 1a). Under the chosen experimental conditions, the environmental isolates grew faster than the human-associated ones (*p* < 0.001; U test). The shift in the median *μ* corresponds to a difference in doubling times of about four minutes. The variability within the two groups does not differ notably from each other (*p* ≈ 0.3; F test).

### Plasmid transfer rates

The observed plasmid transfer rates reasonably follow a log-normal distribution with considerable variance. Averaged over all isolates, the width of the interquartile range is about one log unit; the range between 5 and 95 percentile representing 90% of the isolates covers more than three orders of magnitude (10^−15.4^ to 10^−12.0^ mL cells^–1^ hour^–1^). The observed extremes are 10^−16.3^ and 10^−11.5^, respectively.

The average plasmid transfer rates fall in the range from 10^–13^ to 10^–14^ mL cells^–1^ hour^–1^ without significant differences between the two subsets of isolates (Fig 1b). However, the variance among human-associated *E. coli* proved to be higher compared to the environmental isolates (*p* < 0.01; F test).

Numerical values of the plasmid transfer rates for the selected recipients from Table 1 can be found in S1 Table (bold numbers).

### Impact of low antibiotic exposure on plasmid transfer rates

**Fig 2:**
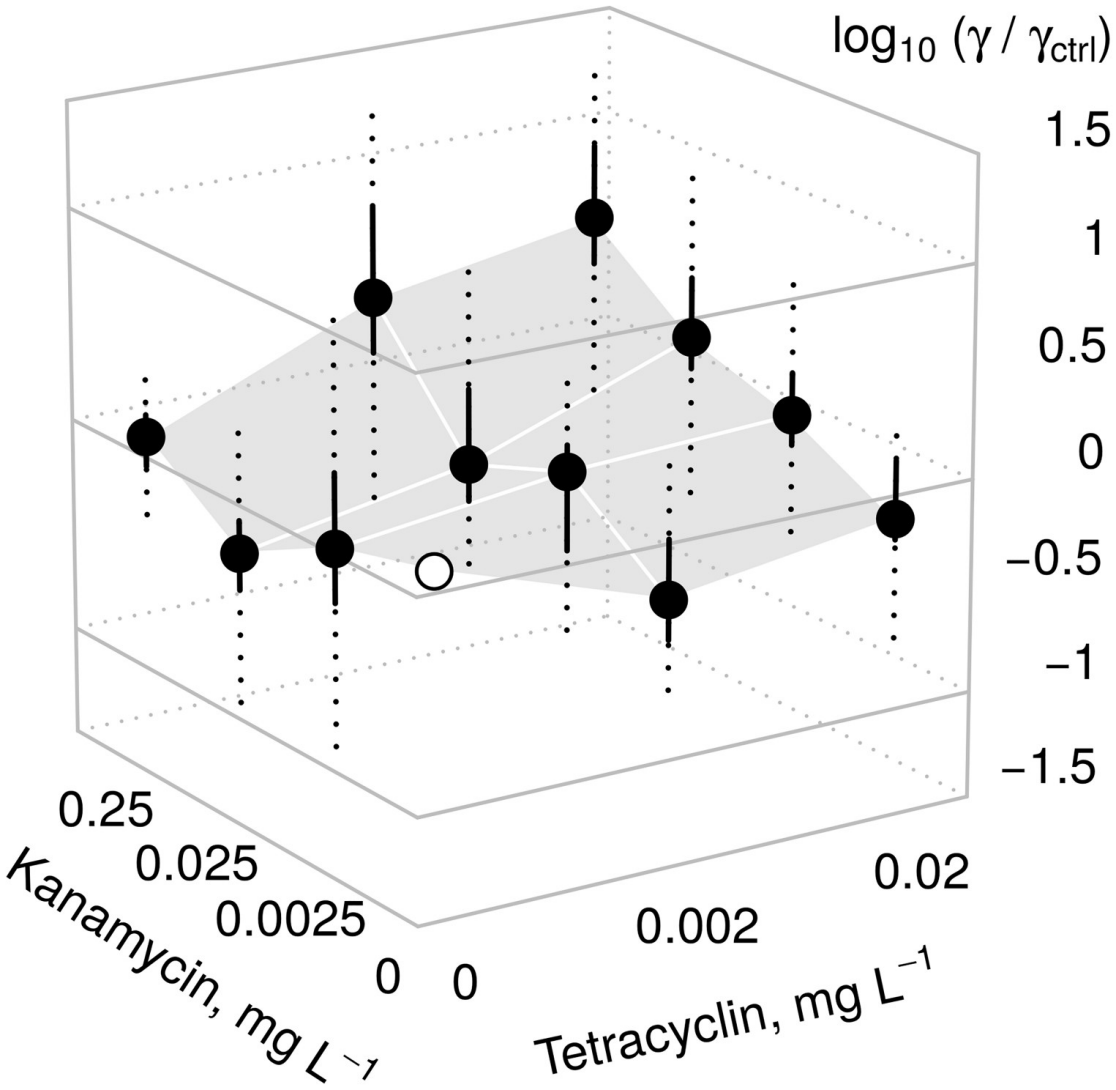
Response of the bulk conjugation rate constant *γ* to sub-inhibitory levels of antibiotics. The control experiment without any antibiotics (*γ* = *γ*_ctrl_) is marked by an empty circle. One unit on the vertical axis corresponds to an increase (positive) or decrease (negative) of *γ* by a factor of 10. Filled dots represent the average change, computed as the median over the 16 tested strains. Solid lines represent interquartile ranges and dashed lines extend to the observed extremes.

Within the considered range of concentrations, antibiotic exposure only led to a moderate change in plasmid transfer rate constants (Fig 2). The most extreme response observed was an increase of *γ* by factor 4 (averaged over the 16 tested isolates) or factor 15 (for a particular isolate), respectively. Considering the response averaged over the tested recipients (bullet symbols in Fig 2), the data suggest a general increase in *γ* when the two antibiotics are applied in combination at higher dosages (log_10_ *γ*/*γ*_ctrl_ > 0). The increase was observed for both the environmental *E. coli* (*p* < 0.05; one-sample t test) as well as for the human-associated isolates (*p* < 0.01). With regard to the shape of the response surface illustrated in Fig 2, no systematic differences were detected between these two subsets of isolates. The values of log_10_ *γ*/*γ*_ctrl_ did not differ significantly between the subsets at any point in concentration space (two-sample *t* tests).

Numerical values of the transfer rates for the selected recipients from Table 1 can be found in S1 Table.

## 1 Discussion

Our experimental results demonstrate a huge inter-strain variability with respect to the uptake of an AR-carrying plasmid by *E. coli*. Conjugative plasmid transfer rates differed by up to about four orders of magnitude between the most efficient and the poorest *E. coli* recipient. This range is in accordance with experimental data presented by [6] who studied the conjugation capabilities of *E. coli* isolates with a background in humans and other mammals.

Since the donor (*Serratia marcescens*) and the recipient *E. coli* exhibit a close phylogenetic relation, we could not fully exclude a priori that either strain was inhibited in the mating assays. Inhibition effects might invalidate the strain-specific growth rate constants observed in single-strain cultures and thus compromise the estimates of *γ* [16]. We explicitly tested a subset of four mating pairs (two with hight *γ* and two with low *γ*) for mutual inhibition of growth. We did not find evidence for suppressed growth of the donor in presence of recipients. Regarding the latter, plating-based cell counts suggest only minor inhibition effects. On average, the apparent growth rate constants of recipients were reduced by 13% compared to single-strain assays (max. 21%). According to a model-based sensitivity analysis, mis-estimates in the growth rate constant of this magnitude would translate into a negative bias in *γ* of 1/4 log unit. The large inter-strain variation in *γ* of up to four log units (Fig 1b) is thus very unlikely to be an artifact rooted in the neglect of inhibition between donors and recipients.

The contrasting growth rates of environmental and human-associated isolates (Fig 1a) suggest that river sediments and wastewater harbor distinct populations of *E. coli*. It is plausible to assume that the wastewater population is dominated by human-borne isolates while the sediment population is rather recruited from strains with a history in the guts of wild animals or livestock. On the one hand, these two populations might be stabilized by the continuous inoculation from contrasting sources of bacteria (i.e. human waste and runoff). On the other hand, active growth of *E. coli* under environmental conditions has been demonstrated [29] and seemingly stable populations have been found in secondary habitats [30–32]. Thus, the difference in growth characteristics found here (Fig 1a) could also reflect diverging selection pressures in the two habitats. Specifically, the higher growth rates of the sediment-borne isolates might be a result of positive selection for strains adapted to the utilization of complex, low quality carbon sources as contained in the chosen medium (HT broth).

Based on the contrasting growth rates of environmental and human-associated isolates, we anticipated to also find differences between the two groups of isolates in terms of their capabilities for plasmid uptake. Contrary to expectation, plasmid transfer occurred at similar rates regardless of the recipient strain’s origin (Fig 1b). Consequently, we have to stick to the null hypothesis according to which environmental isolates of *E. coli* have a similar potential for the uptake of plasmid-borne resistance genes as their human-associated counterparts.

**Fig 3:**
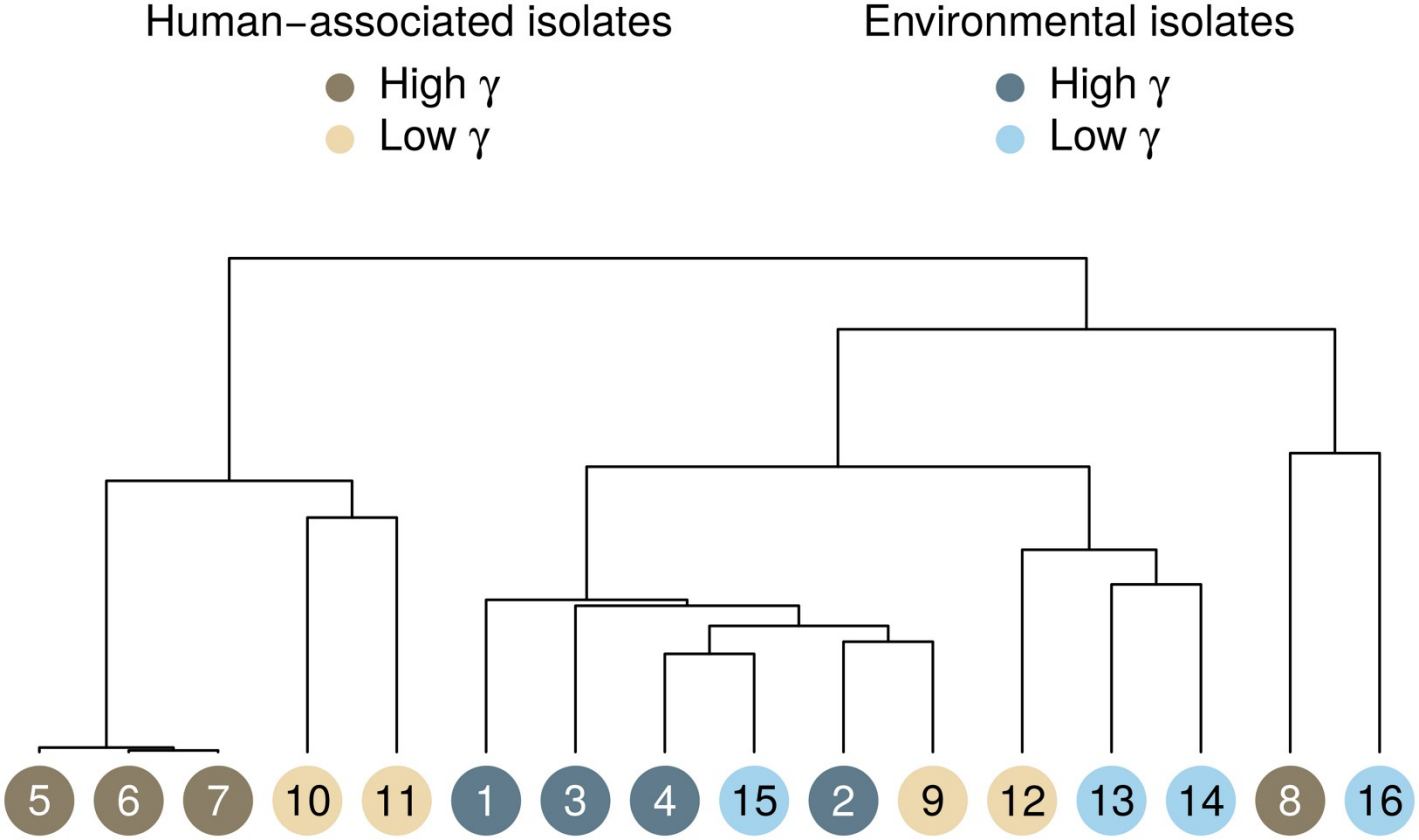
Dissimilarity of the 16 selected *E. coli* recipients based on their genetic equipment. Identifiers 1–16 correspond to Table 1 and S1 Table. Colors indicate the origin of the isolates and color intensity codes for the efficiency of plasmid uptake. The separation between high and low *γ* was taken to be the average value of all strains.

In order to shed more light on the peculiarities of environmental and human-associated *E. coli* strains, respectively, eight isolates from each group were whole-genome sequenced and genetic dissimilarities were explored with hierarchical cluster analysis. While five of the human-associated isolates formed a separate group at top-level, the remaining three isolates clustered together with *E. coli* of environmental origin (Fig 3). This outcome suggests that environmental and human-borne isolates do not form two phylogenetically homogeneous groups but partial mixing occurs, e.g. in response to anthropogenic river pollution. Such heterogeneity might obfuscate actual differences between the two groups in terms of plasmid uptake efficiency and it provides a possible explanation for the lack of significance in Fig 1b. Nevertheless, the diverging growth rates (Fig 1a) indicate that differences between the environmental and human-associated population remain in spite of partial mixing.

In summary, we conclude that differences between the two populations manifest themselves primarily in the efficiency of growth on a complex carbon source and to a much lesser extend in the efficiency of plasmid uptake under the chosen experimental conditions.

Previous research has pointed out the importance and complexity of plasmid-plasmid interactions [33]. Already [6] proposed that the ability of plasmid uptake might be related to the presence of further compatible plasmids in the recipient. We did not find evidence for such a relation based on the genome analysis of the 16 strains from Table 1. The value of *γ* was neither significantly correlated with the number of harbored plasmids nor was it associated with the presence of a particular plasmid (t test). Likewise, *γ* appeared to be unrelated to the number of identified virulence factors. Because of the limited data set, however, it is possible that existing relations remained undetected.

Previous research has examined the overall effect of drug exposure on the spread of antibiotic resistance without differentiating between vertical and horizontal gene transfer [34–36]. By contrast, we explicitly studied the influence of antibiotic exposure on the rate constant of plasmid-mediated *horizontal* gene acquisition. The latter aspect has only been addressed by few researchers and results are heterogeneous. For example, [37] did not find evidence for a direct control of conjugative gene transfer by sub-lethal concentrations of several antibiotics. By contrast, [38] demonstrated the upregulation of genes involved in RP4 replication and mating pair formation in response to low ambient concentrations of carbamazepine (an antiepileptic drug).

Our data suggest that antibiotic exposure can in fact trigger changes in plasmid transfer rates which are significant in the statistical sense. However, the moderate alteration of *γ* induced by antibiotic exposure is likely to be of minor relevance in real-world settings where bacterial populations consist of multiple strains with largely contrasting capabilities for plasmid uptake (Fig 1b). In multi-strain populations, the overall efficiency of plasmid uptake would clearly be dictated by the proportions of individual strains rather than by the effects of low-level antibiotic exposure.

## Conclusions

To predict the spread of AR in bacterial populations, reliable information on plasmid transfer rates is needed. Our results suggest that plasmid uptake rates for RP4 are similar among *E. coli* isolated from river sediments and human-borne raw sewage, respectively. The rate constant of plasmid uptake was shown to follow a log-normal distribution with considerable variance among isolates. Even minor shifts in the strains’ relative abundances may therefore strongly alter the net plasmid transfer at population level. In light of that, deterministic modeling strategies relying on a population-averaged rate constants are inadequate and should be augmented or replaced by, e.g., agent-based approaches.

## Supporting information

S1 Table Conjugation rate constants (mL cells^–1^ hour^–1^; log_10_) for the recipients that underwent whole-genome sequencing. Reported are 95% confidence intervals (best-fit value ±2 × standard error). Bold numbers refer to the control experiment without antibiotic exposure.
(PDF)
