# Supplementary material for: High variability of plasmid uptake rates in Escherichia coli isolated from sewage and river sediments
Source: PLoS One. 2020 Apr 30;15(4):e0232130. doi: 10.1371/journal.pone.0232130 (PMC7192377; doi:10.1371/journal.pone.0232130)
Supplement: S1 Table — Reported are 95% confidence intervals (best-fit value ±2 × standard error). Bold numbers refer to the control experiment without antibiotic exposure. (PDF) [file pone.0232130.s001.pdf]

Conjugation rate constants (mL cells^{-1} hour^{-1}; $\log_{10}$) for the recipients that underwent whole-genome sequencing. Reported are 95% confidence intervals (best-fit value ± 2 × standard error). Bold numbers refer to the control experiment without antibiotic exposure.

	Tetracyclin	Kanamycin (mg L^{-1})			
Isolate	(mg L^{-1})	0	0.0025	0.025	0.25
1	0	**-12.8 ± 0.1**	-12.8 ± 0.2	-13.3 ± 0.1	-12.9 ± 0.1
	0.002	-13.1 ± 0.1	-12.6 ± 0.1	-12.7 ± 0.1	-12.7 ± 0.1
	0.02	-12.7 ± 0.1	-12.5 ± 0.1	-12.6 ± 0.1	-12.2 ± 0.1
2	0	**-12.2 ± 0.1**	-11.4 ± 0.1	-13 ± 0.1	-12.4 ± 0.1
	0.002	-12.2 ± 0.1	-12.8 ± 0.2	-12.4 ± 0.1	-12 ± 0.1
	0.02	-12.7 ± 0.2	-12.1 ± 0.1	-11.9 ± 0.1	-11.7 ± 0.1
3	0	**-12.8 ± 0.1**	-12.2 ± 0.1	-13.1 ± 0.1	-12.9 ± 0.1
	0.002	-12.5 ± 0.1	-12.7 ± 0.1	-13.2 ± 0.1	-12.7 ± 0.1
	0.02	-12.7 ± 0.1	-12.8 ± 0.1	-12.3 ± 0.1	-12.1 ± 0.1
4	0	**-12.6 ± 0.1**	-11.7 ± 0.1	-13.3 ± 0.1	-13 ± 0.1
	0.002	-13.1 ± 0.2	-13.3 ± 0.1	-12.3 ± 0.1	-12.2 ± 0.1
	0.02	-13.4 ± 0.1	-12.4 ± 0.2	-12.1 ± 0.1	-12.2 ± 0.2
5	0	**-12.2 ± 0.1**	-12.7 ± 0.1	-13.7 ± 0.2	-12.2 ± 0.1
	0.002	-13 ± 0.1	-11.9 ± 0.1	-12.1 ± 0.1	-11.3 ± 0.1
	0.02	-12.7 ± 0.1	-12 ± 0.1	-12.5 ± 0.1	-10.8 ± 0.1
6	0	**-12.5 ± 0.1**	-12.4 ± 0.1	-13.6 ± 0.2	-12.5 ± 0.1
	0.002	-12.4 ± 0.1	-12.3 ± 0.1	-11.7 ± 0.1	-11.2 ± 0.1
	0.02	-12.8 ± 0.1	-11.8 ± 0.1	-11.2 ± 0.1	-11.2 ± 0.1
7	0	**-12.1 ± 0.1**	-12.2 ± 0.2	-13.6 ± 0.1	-12.5 ± 0.1
	0.002	-12.7 ± 0.1	-12.3 ± 0.1	-12.8 ± 0.1	-11.3 ± 0.1
	0.02	-12.4 ± 0.1	-11.4 ± 0.1	-11.3 ± 0.1	-11 ± 0.1
8	0	**-12.9 ± 0.1**	-12 ± 0.2	-13.3 ± 0.1	-12.7 ± 0.1
	0.002	-13 ± 0.1	-12.9 ± 0.1	-12.3 ± 0.1	-12.2 ± 0.1
	0.02	-13.2 ± 0.1	-12.7 ± 0.1	-12.4 ± 0.1	-11.8 ± 0.2
9	0	**-13.6 ± 0.1**	-14.6 ± 0.1	-14.2 ± 0.1	-13.8 ± 0.1
	0.002	-14.2 ± 0.1	-14.3 ± 0.1	-13.9 ± 0.1	-13 ± 0.1
	0.02	-13.8 ± 0.1	-14.1 ± 0.1	-13.3 ± 0.1	-13 ± 0.1
10	0	**-14.1 ± 0.1**	-14.1 ± 0.1	-13.9 ± 0.1	-13.6 ± 0.1
	0.002	-14.4 ± 0.1	-13.5 ± 0.2	-13.6 ± 0.1	-12.8 ± 0.1
	0.02	-14 ± 0.1	-13.8 ± 0.1	-13.1 ± 0.1	-13.2 ± 0.1
11	0	**-14.2 ± 0.1**	-14.4 ± 0.1	-14.5 ± 0.1	-13.9 ± 0.1
	0.002	-14.4 ± 0.1	-14.1 ± 0.1	-14.8 ± 0.1	-13.8 ± 0.1
	0.02	-14.1 ± 0.1	-14.4 ± 0.1	-14 ± 0.1	-13.8 ± 0.1
12	0	**-14.4 ± 0.1**	-14.7 ± 0.1	-14.8 ± 0.1	-14.3 ± 0.1
	0.002	-14.9 ± 0.1	-14.4 ± 0.1	-14.8 ± 0.1	-15 ± 0.1
	0.02	-14.2 ± 0.1	-14.5 ± 0.1	-14.3 ± 0.1	-14.5 ± 0.1
13	0	**-14 ± 0**	-14.2 ± 0.1	-14.4 ± 0.1	-14 ± 0.1
	0.002	-14.1 ± 0.1	-13.9 ± 0.1	-14.3 ± 0.1	-14 ± 0.1
	0.02	-13.9 ± 0.1	-13.9 ± 0.1	-14.5 ± 0.1	-14.3 ± 0.1
14	0	**-13.4 ± 0**	-13.8 ± 0.1	-13.6 ± 0.1	-13.6 ± 0.1
	0.002	-13.8 ± 0.1	-14.2 ± 0.1	-13.6 ± 0.1	-13 ± 0.1
	0.02	-13.6 ± 0.1	-13.5 ± 0.1	-13.4 ± 0.1	-13.1 ± 0.1
15	0	**-13.5 ± 0**	-14.2 ± 0.1	-13.3 ± 0.1	-13.5 ± 0.1
	0.002	-14.2 ± 0.1	-13.8 ± 0.2	-13.7 ± 0.1	-12.2 ± 0.1
	0.02	-13.9 ± 0.1	-13.6 ± 0.1	-13.4 ± 0.1	-12.9 ± 0.1
16	0	**-14.1 ± 0.1**	-14.6 ± 0.1	-13.9 ± 0.1	-14.1 ± 0.1
	0.002	-14.6 ± 0.1	-14.1 ± 0.1	-14.5 ± 0.1	-14.1 ± 0.1
	0.02	-14.1 ± 0.1	-14.2 ± 0.1	-14.1 ± 0.1	-13.9 ± 0.1
